# Efficacy of thiamine (vitamin B1) in sepsis and septic shock: A meta-analysis of randomized controlled trials

**DOI:** 10.1016/j.clinsp.2026.100901

**Published:** 2026-03-20

**Authors:** Xiaoqin Xu, Xiaoqi Bai, Wei Cao, Danting Fei, Dandan Yang, Xuning Shen, Jun Xu, Dongjun Xu

**Affiliations:** Department of Emergency Medicine, Affiliated Hospital of Jiaxing University, The First Hospital of Jiaxing, Jiaxing, Zhejiang 314001, China

**Keywords:** Sepsis, Septic shock, Thiamine, Mortality, Renal replacement therapy

## Abstract

**Objective:**

To perform a meta-analysis evaluating thiamine’s efficacy for sepsis and septic shock to support its clinical use.

**Methods:**

The meta-analysis of randomized controlled studies on patients with sepsis and septic shock evaluated the efficacy of intravenous thiamine. Articles were retrieved from online databases. Qualities of included studies were assessed.

**Results:**

Nine studies enrolling 520 patients were included. Regarding bias risk assessment of studies, two articles were classified as low risk, three were categorized as raising some concerns, and four were identified as high risk. Patients in the thiamine group were significantly associated with decreased proportion of Renal Replacement Therapy (RRT, Odds Ratio [OR = 0.26], *p* = 0.0007), decreased 24 h lactate level (Mean Difference [MD = -0.35], *p* = 0.004), and larger 24 h SOFA score change (MD = 1.15, *p* < 0.00001). No statistical significance was detected in short-time mortality (OR = 0.78, *p* = 0.18) and 24 h lactate change (MD = 0.09, *p* = 0.36) between the two groups. In the thiamine-deficient subgroup (2 studies, 51 patients), exploratory analysis indicated a potential short-term mortality reduction with thiamine (OR = 0.18, *p* = 0.01), though limited by the small sample size.

**Conclusion:**

This meta-analysis preliminarily suggested that thiamine may exert a potential role in improving organ dysfunction among patients with sepsis and septic shock. No significant benefit of thiamine in reducing short-term mortality was observed. It is important to note that the total number of patients included in this meta-analysis is relatively small, which limits the statistical power and external validity of the results.

## Highlights

• Meta-analysis evaluates thiamine’s efficacy for sepsis/septic shock.
• Meta-analysis includes RCTs on intravenous thiamine for sepsis/septic shock.
• Thiamine reduces the need for Renal Replacement Therapy (RRT).
• Thiamine increases 24 h SOFA score changes and improves organ dysfunction.
• Overall short-term mortality unchanged; subgroup has lower mortality.

## Introduction

Sepsis, an acute life-threatening organ dysfunction syndrome characterized by dysregulated response to infection, can cause sequential multiple organ dysfunction and ultimately death.^1^ There are approximately 48.9 million newly diagnosed cases of sepsis worldwide, among which about 11 million cases result in death, accounting for 19.7 % of total global deaths.^2^ Fluid resuscitation, early administration of antibiotics and vasopressors constitute the foundation of sepsis treatment.^3^^,^^4^ With the utilization of the sepsis early warning system and the implementation of the sepsis bundle therapy, the mortality rate of sepsis has declined^4^^,^^5^ however, it still constitutes a major cause of global health loss^2^ The expense of treating sepsis is quite high.^6^ Even after being discharged, patients experience long-term health consequences, leading to their inability to return to work.^7^

Thiamine, also known as vitamin B1, is a cofactor for various cellular enzymes and these enzymes are of vital importance in aerobic carbohydrate metabolism, maintenance of the cellular redox state, mitochondrial oxidative phosphorylation, and the synthesis of adenosine triphosphate.^8^ Thiamine deficiency is a relatively common condition observed in patients suffering from sepsis.^9^^,^^10^ A retrospective study showed that early supplement of thiamine can reduce 28-day mortality in septic shock.^11^ In a recently published prospective study, no benefits regarding mortality were observed in septic shock patients receiving thiamine.^12^^,^^13^ Considering inconsistent findings, Guidelines for management of sepsis and septic shock 2021 made no recommendation regarding thiamine.^14^ Recently published Randomized Controlled Studies (RCTs) reported discrepant results,^12^^,^^13^^,^^15^ so it is necessary to perform a meta-analysis and re-evaluate the effectiveness of thiamine in sepsis and septic shock.

## Methods

The protocol for this systematic review and meta-analysis was officially recorded in PROSPERO (registration number: CRD42025631047) on Jan 17, 2025. Our review adhered to the guidelines of Preferred Reporting Items for Meta-analyses (PRISMA) and followed the Cochrane manual for systematic reviews.^16^^,^^17^

### Eligibility criteria

Clinical trials fulfilling all the following criteria were included: 1) Study type: RCTs, 2) Population: adult patients diagnosed with sepsis or septic shock according to Sepsis 1.0,^18^ Sepsis 2.0,^19^ or Sepsis 3.0^20^ definitions, 3) Intervention: IV thiamine (vitamin B1) alone, 4) Control: placebo or no additional intervention apart from standard treatment, 5) Outcome: short time mortality, receipt of Renal Replacement Therapy (RRT), lactate at 0 and 24 h, SOFA score at 0 and 24 h, and 6) English language. The exclusion criteria were summarized below: 1) Studies on non-infectious disease and on vitamin C treatment; 2) Participants who received combination treatment (thiamine plus hydrocortisone, thiamine plus vitamin C, etc.); 3) Repeated studies; 4) Single-arm trials, observational studies, conference proceedings, case reports, review and meta-analyses, and non- human studies; 5) Articles unable to yield needed information.

### Searching databases

Clinical trials were sourced from five distinct databases (PubMed, Web of Science, Scopus, EMBASE, and Cochrane) up until Dec 25, 2024. The keywords below were adopted in our meta-analysis: sepsis, septic shock, bloodstream infection, thiamine, vitamin B1 and aneurine.

### Studies selection

Articles downloaded from databases were imported into Zotero software and the process of removing repeated articles was performed by the software. A tripartite selection procedure was used. Firstly, titles and abstracts of all studies were assessed to exclude obviously unsuitable ones. Secondly, an examination of full texts of remaining studies was conducted based on eligibility criteria to pick out relevant articles. Thirdly, a hands-on examination of the references from pertinent trials was done to trace all related trials. Two researchers independently conducted each phase. When there were discrepancies between the two researchers, a senior researcher joined and discussed with them until an agreement could be reached. The authors of articles that were included in the review were contacted to get any desired information if necessary.

### Data extraction

Information from eligible studies was retrieved via Excel independently by two researchers. Study protocols, clinical trial registration websites, published full texts, and inquiries from the authors were sources of information. Two researchers read the article together and discussed carefully to settle any discrepancies. General characteristics of the enrolled trials and information on outcomes were extracted, which consisted of the first author’s name, the year of publication, grouping, the number of patients, ages, details of intervention, comorbidities, laboratory values, clinical scores, mortality, proportion of RRT, duration of vasopressors, duration of mechanical ventilation and so on.

### Outcome measure

Short-term mortality was chosen as the primary outcome, which was defined as in-hospital, 28-day or ICU mortality depending on the availability. The secondary outcomes were the 24 h lactate change, the 24 h lactate level, the 24 h SOFA score change, adverse events, and the proportion of RRT. The 24 h lactate change was defined as the lactate level at enrollment minus the lactate level at 24 h; similarly, this definition applied to the 24 h SOFA score change.

### Quality assessment

Two researchers evaluated the risk of bias based on methodological quality of the studies using the latest Version 2 of the Cochrane risk-of-bias tool for randomized trials (RoB2).^21^ When inconsistent results of risk of bias arose, a third researcher joined the discussion and reviewed the full article. This continued until all disagreements were dismissed. If information from online materials was not enough to reach a conclusion on quality assessment, the authors communicated with other authors to ask for the required details.

### Data analysis

Data analysis was performed using RevMan Web (version: 7.1.2) and Stata Software Package (version 17.0). The Mean Difference (MD) and the 95 % Confidence Interval (95 % CI) were calculated for continuous outcomes using the inverse variance method. The Odds Ratio (OR) and 95 % CI were calculated for binary outcomes using the Mantel-Haenszel method; p-value of less than or equal to 0.05 was considered statistically significant. When median and quartiles were presented as summary statistics for outcomes, the quantile estimation method was used to calculate the mean and standard deviation.^22^, ^23^, ^24^ The heterogeneity of outcomes was evaluated based on the Chi-Square test and the *I*^2^ index. If p ˂ 0.1 with *I*^2^ > 50 %, indicating heterogeneous, a random-effect model was chosen as the analysis model. Otherwise, the outcome was regarded as homogeneous, and a fixed-effect model was chosen. Subgroup analysis was conducted in the thiamine deficiency group. Sensitivity analysis was conducted by excluding a study in turn to explore the stability of the pooled estimates and possible sources of heterogeneity. Publication bias was evaluated for the primary and secondary outcomes by Egger’s test.

## Results

### Summary of outcomes from searching databases and studies selection

**Fig. 1:**
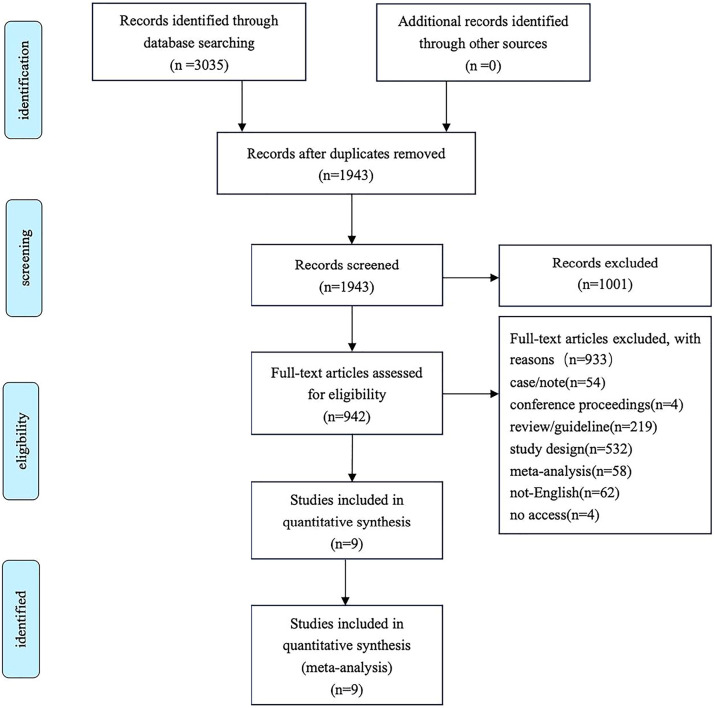
Flowchart of study selection.

The authors identified 3035 articles up until December 25, 2024 from 5 databases and other sources (Fig. 1). After basic screening of obviously unsuitable studies, 942 studies underwent full-text reading. Ultimately, a total of 9 studies^12^^,^^13^^,^^15^^,^^25^, ^26^, ^27^, ^28^, ^29^, ^30^ was incorporated into our meta-analysis following the exclusion of ineligible studies for various reasons. Fig. 1 presents the PRISMA flowchart detailing the process of database searching and study selection. Of the 9 studies, 2 studies^28^^,^^30^ reported the same clinical trial but disclosed different sections of data. Therefore, there were 8 clinical trials enrolled in the review. Of the 8 trials, 2 were conducted in the USA,^12^^,^^28^ 2 in India,^15^^,^^25^ 1 in Thailand,^26^ 1 in Indonesia,^29^ 1 in Malaysia^27^ and 1 in Brazil.^13^

### Characteristics of the included patients

Our review encompassed 520 patients from 8 RCTs, with 256 patients undergoing treatment with thiamine and 264 patients receiving placebo treatment. The minimum sample size for a clinical trial was 24 and the maximum sample size was 115. Seven clinical trials provided information on ages and the mean age of patients ranged from 44- to 74-years in the thiamine group and 48- to 70-years in the control group. As for the dosage regimen of thiamine, 231 patients in 7 trials^12^^,^^13^^,^^25^, ^26^, ^27^, ^28^, ^29^ received 200 mg of thiamine intravenously twice a day for various durations, while 28 patients in a trial^15^ received 2 mg/kg of thiamine intravenously 8 hourly for 72 h. One hundred and eleven patients in 4 trials^12^^,^^15^^,^^27^^,^^29^ received thiamine for 3-days, 77 patients in 2 trials^13^^,^^25^ for 5-days, and 68 patients in 2 trials^26^^,^^28^ for either 7-days or until discharge. The SOFA score varied from 3.2 to 13.5 in the experimental group and from 3.3 to 12.5 in the control groups. Summary of characteristic of studies and patients was outlined in Table 1.

**Table 1:** Characteristics of patients in included studies.

Study ID	Thiamine group					Control group				
	N	Age, mean /SD	Male	SOFA, mean /SD	Methods	N	Age, mean / SD	Male	SOFA, mean / SD	**Methods**
Ap 2022	20	NA	10	NA	200 mg, twice, intravenous for 5 days	20	NA	16	NA	Placebo
Donnino 2016	43	70/14	26	10.1/3.7	200 mg, twice, 7 days or until discharge	45	65/17	26	10.2/3.7	Placebo
Harun 2019	32	61/16	20	13.5/2.3	200 mg, twice, intravenous for 3 days	33	66/17	18	12.5/2.7	Placebo
Moskowitz 2017	31	68/16	18	9.3/3.3	200 mg, twice, 7 days or until discharge	39	66/17	22	9.9/3.7	Placebo
Moskowitz 2023	42	74/15	18	11.3/3.6	200 mg, twice, intravenous for 3 days	46	70/13	27	11.5/3.8	Placebo
Nandhini 2022	25	54/17	15	12.2/0.9	2 mg/kg, 4 times a day, intravenous for 3 days	25	54/15	11	12.2/1.1	Placebo
Nasution 2020	12	44/15	NA	3.2/0.5	200 mg, twice, intravenous for 3 days	12	48/16	NA	3.3/1.6	Placebo
Pereira 2023	57	63/16	27	9.5/3.6	200 mg, twice, intravenous for 5 days	58	65/13	36	10/3.4	Placebo
Petsakul 2020	25	64/20	17	10/3.9	200 mg, twice, 7 days or until discharge	25	66/17	12	11/2.5	Placebo

SOFA, Sequential Organ Failure Assessment; NA, Not Available.

### Quality assessment results

**Fig. 2:**
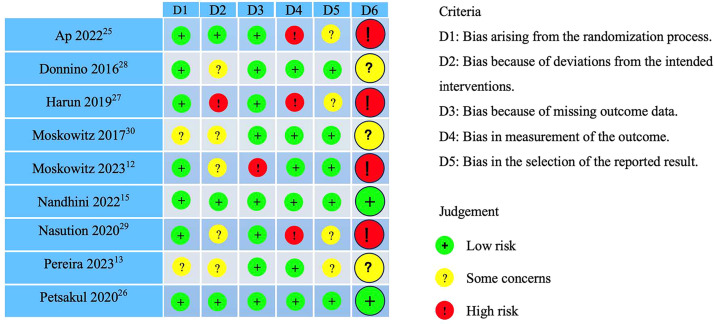
Risk of bias assessment.

Fig. 2 illuminated the results of the Risk of Bias Assessment (RoB2) of 9 included articles. In terms of overall risk assessment, 2 articles were classified as low risk,^15^^,^^26^ 3 articles raised some concerns,^13^^,^^28^^,^^30^ and 4 articles were categorized as high risk.^12^^,^^25^^,^^27^^,^^29^ Of the 3 articles ranked as having some concerns, one article^28^ raised concerns regarding deviations from the intended interventions. Another article^30^ identified risks associated with the randomization process and deviations from the intended interventions. The third article^13^ highlighted risks related to the randomization process, deviations from the intended interventions, and the selection of reported results. Of the 4 articles identified as high risk, one article^25^ did not clarify whether the outcome evaluator was aware of the intervention modalities, which raised concerns about their ability to assess the objectivity of the results; The second article^27^ highlighted risks related to deviations from the intended interventions and measurement of the outcome; The third article^12^ was doubted because of deviations from the intended interventions; The forth article^29^ was labeled high risk of bias due to impropriety in measurement of the outcome.

### Primary outcomes

**Fig. 3:**
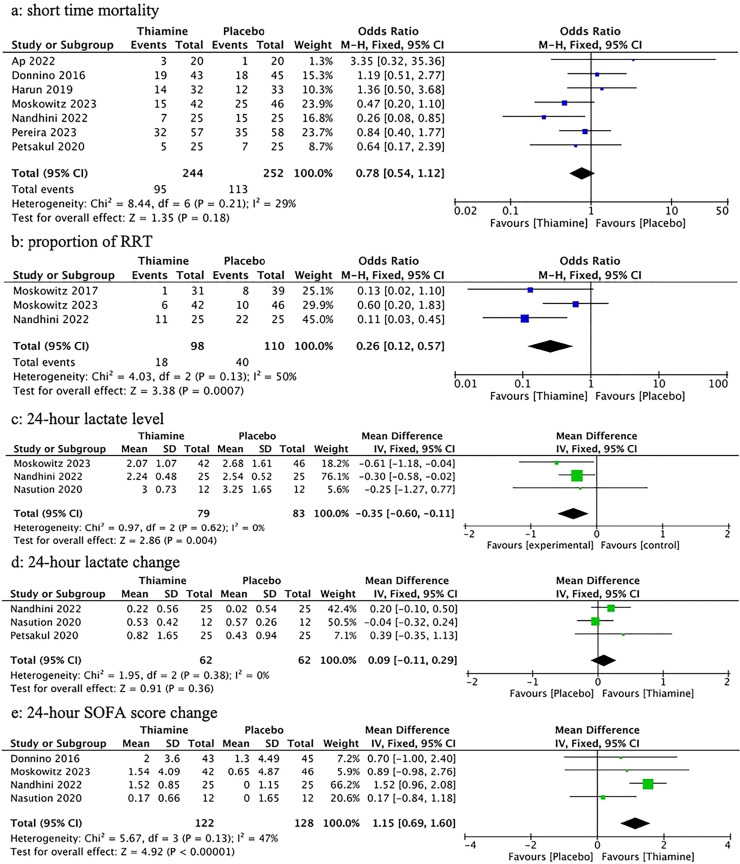
Forest plots for primary and second outcomes (a) Short time mortality, (b) Proportion of RRT, (c) 24 h lactate level, and (d) 24 h lactate change, (e) 24 h SOFA score change. RRT, Renal Replacement Treatment; SOFA, Sequential Organ Failure Assessment; CI, Confidence Interval; df, degrees of freedom; MH, Mantel-Haenszel; IV, Inverse Variance; SD, Standard Deviation.

Seven studies^12^^,^^13^^,^^15^^,^^25^, ^26^, ^27^, ^28^ involving 496 patients reported the short-term mortality outcome, which occurred in 208 patients altogether. Ninety-five patients in the thiamine group expired (mortality rate: 37.1 %); One hundred and thirteen patients in the control group expired. Intravenous administration of thiamine did not demonstrate a statistically significant decrease in short-term mortality (OR=0.78, 95 % CI 0.54–1.12, *p* = 0.18; Fig. 3a). No important heterogeneity was observed across studies (*p* = 0.21, I^2^=29 %).

### Secondary outcomes

#### The proportion of RRT

The result was documented in 3 studies^12^^,^^15^^,^^30^ Pooled results showed that thiamine treatment significantly reduced receipt of RRT in patients with sepsis and septic shock (18.4 % vs. 36.4 %, OR = 0.26, 95 % CI 0.12−0.57, *p* = 0.0007; Fig. 3b). The outcome showed no important heterogeneity (*p* = 0.13, *I*^2^ = 50 %).

#### The 24 h lactate level

The 24 h lactate levels of 166 patients in 3 trials^12^^,^^15^^,^^29^ were analyzed. Patients in the intervention group had a significantly lower level of 24 h lactate compared to those in the control group (MD = -0.35, 95 % CI -0.60 to -0.11, *p* = 0.004; Fig. 3c). The data were homogenous (*p* = 0.62, *I*^2^ = 0 %).

#### The 24 h lactate change

Three studies^15^^,^^26^^,^^29^ with 124 patients revealed the data on the 24 h lactate change, which could be converted into Mn/SD and analyzed in the meta-analysis. There was no statistical difference (MD = 0.09, 95 % CI -0.11 to 0.29, *p* = 0.36; Fig. 3d) between the thiamine and placebo groups in terms of the 24 h lactate change. Heterogeneity across studies was regarded as low (*p* = 0.38, *I*^2^ = 0 %).

#### The 24 h SOFA score change

Data on the 24 h SOFA score change were extracted in 250 patients from 4 studies.^12^^,^^15^^,^^28^^,^^29^ The pooled result indicated that patients in the thiamine group exhibited a more significant change in the 24 h SOFA score compared to those in the control group, and this difference was statistically significant (MD = 1.15, 95 % CI 0.69 to 1.60, *p* < 0.00001; Fig. 3e). Important heterogeneity was not detected across studies (*p* = 0.13, *I*^2^ = 47 %).

#### Adverse events

Only one study^12^ reported the adverse events and no serious adverse events related to the study drug occurred.

### Publication bias and sensitivity analysis

Publication bias was assessed for the primary and secondary outcomes through Egger’s test. The results of Egger’s test showed no statistically significant publication bias (Table S1). Sensitivity analyses were done for primary and secondary outcomes by excluding a study in turn. All the outcomes were robust except the outcome of the 24 h SOFA score change, which was disproportionately influenced by a trial.^15^ Plots of results of the sensitivity analysis were shown in Supplemental Material (Figs. S1–S5).

### Subgroup analysis

**Fig. 4:**
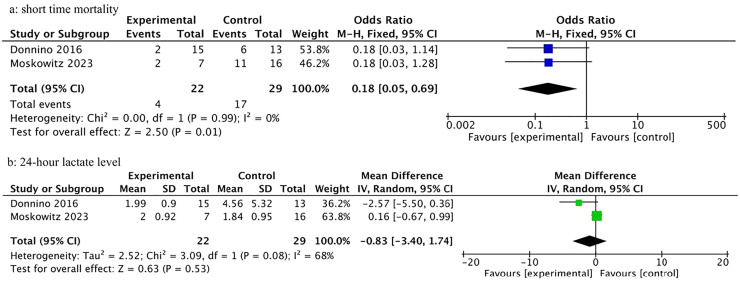
Forest plots for the outcomes of subgroup analysis (a) Short time mortality, (b) 24 h lactate level; df, degrees of freedom; MH, Mantel-Haenszel; IV, Inverse Variance; SD, Standard Deviation.

Subgroup analyses based on the concentration of thiamine in the blood were performed. Only data on the thiamine deficient patients could be extracted and analyzed. A post-hoc subgroup analysis of two trials (*n* = 51) in thiamine-deficient patients suggested short-term mortality was significantly reduced (18.2 % vs. 58.6 %, OR = 0.18, 95 % CI 0.05−0.69, *p* = 0.01; Fig. 4a), yet they had no impact on 24 h lactate levels (MD = -0.83, 95 % CI -3.40 to 1.74, *p* = 0.53; Fig. 4b). There was heterogeneity in the lactate level outcome (*p* = 0.08, *I*^2^ = 68 %) but not in the mortality outcome (*p* = 0.99, *I*^2^ = 0 %).

## Discussion

This meta-analysis of nine studies (with a total sample size of 520 patients) indicated that thiamine may have a potential role in improving outcomes for patients with sepsis and septic shock, specifically in reducing the need for renal RRT, lowering 24 h lactate levels, and increasing 24 h SOFA score change. It had no effect on short-term mortality or 24 h lactate change. Nevertheless, most of the included studies had very small sample sizes, and a large proportion carried a high risk of bias (four studies were classified as high risk, and three raised some concerns, according to the RoB2 tool). In particular, biases arising from deviations from intended interventions (e.g., inconsistent thresholds for initiating continuous renal replacement therapy) and from outcome measurement (e.g., SOFA components influenced by sedation or clinical judgment, non-standardized timing of lactate sampling) may systematically overestimate the therapeutic effects on clinically driven endpoints. An exploratory subgroup analysis with a limited sample size revealed that thiamine administration showed a potential improvement in mortality among patients with thiamine deficiency. However, this finding can only be regarded as hypothesis-generating rather than hypothesis-confirming evidence; it does, nonetheless, provide insights for future research.

Thiamine is an essential water-soluble vitamin, and deficiency can lead to conditions such as beriberi and Wernicke-Korsakoff syndrome.^31^^,^^32^ Thiamine Pyrophosphate (TPP), as a biochemically active form of thiamine, is an essential cofactor of Pyruvate Dehydrogenase (PDH).^33^^,^^34^ The acetyl coenzyme A produced by catalysis of PDH serves as the substrate for the Krebs cycle.^35^ Thiamine is also an important coenzyme in the pentose phosphate pathway and plays a role in mitigating oxidative stress.^28^^,^^36^ Therefore, a deficiency in thiamine may lead to cellular energy depletion and an excess of reactive oxygen species, ultimately resulting in cell death.^37^

A meta-analysis indicated that the administration of vitamin B1 did not result in an improvement in the short-term mortality in sepsis.^38^ Our study findings are consistent with the aforementioned studies, i.e., the authors did not observe a significant reduction in overall short-term mortality following thiamine administration. The heterogeneity of the mortality outcome was low, and the authors hope that larger-sample studies can support this conclusion. Additionally, the authors did observe improvements in certain secondary outcomes (changes in SOFA scores, lactate levels, and the incidence of renal replacement RRT) following thiamine administration ‒ improvements that were not identified in previous analyses.^38^ This discrepancy may be attributed to the inclusion of new trials and differences in patient populations; for instance, the prevalence of thiamine deficiency was higher in the studies the authors included. A network meta-analysis done by Fujii et al. revealed that thiamine might be detrimental to patients with sepsis.^39^ This may be attributed to the inclusion of combination therapies and a broader sepsis population. By contrast, our focus on thiamine monotherapy may account for the divergent signals regarding organ protection ‒ though our high risk of bias and small sample size limit the ability to draw definitive conclusions. These discrepancies highlight the need for direct head-to-head trials to resolve these uncertainties. Nevertheless, an exploratory subgroup analysis with a limited sample size revealed supplement of thiamine reduced mortality in thiamine-deficient patients, which could result from improvement in renal function and heart function, and a decrease in delirium.^40^, ^41^, ^42^ The sample size included in this outcome analysis is excessively small (a total of 51 cases). As such, the results have significant limitations and should only be regarded as hypotheses for future research, rather than a basis for clinical practice.

The SOFA score was initially designed for the sequential assessment of the severity of organ dysfunction in critically ill patients caused by sepsis and was adopted as an endpoint in clinical trials involving critically ill patients.^43^ It is reported that fixed-day SOFA did not exhibit a significant correlation with mortality, whereas the SOFA change showed a significant association with mortality.^44^ Our meta-analysis suggested that thiamine contributed to a larger decline in 24 h SOFA score, indicating the beneficial effects of thiamine on organ dysfunction. Thiamine could boost cellular energy metabolism, especially in kidneys with abundant mitochondria, and augment the antioxidant capacity of neuronal cells.^45^^,^^46^ These mechanisms may account for the positive outcome, but the outcome was of moderate heterogeneity and should be interpreted with caution.

Sepsis-related kidney injury is not only caused by renal hypoperfusion due to cytokine-mediated vasodilation, but it is also associated with mitochondrial dysfunction.^47^^,^^48^ Thiamine serves as a mitochondrial resuscitator have been investigated by numerous studies in the treatment of septic kidney injury.^12^^,^^30^ In the current meta-analysis, thiamine was related to fewer episodes of RRT. The antioxidant effect of thiamine may contribute to mitigating oxidative stress in kidneys and active TPP can improve the function of mitochondria, thereby reducing the likelihood of acute kidney injury occurring and progressing to the need for RRT.

The elevated serum lactate level indicates a state of organ hypoperfusion.^49^ In cases of sepsis, tissue hypoxia and mitochondrial dysfunction lead to a rapid accumulation of pyruvate, subsequently resulting in metabolic acidosis characterized by hyperlactatemia. The elevated serum lactate level is considered a component of the Sepsis 3.0 definition of septic shock.^50^ Our study revealed that the levels of 24 h lactate were lower in the thiamine group, but the 24 h lactate changes in the two groups were comparable. The decrease in lactate levels may be associated with thiamine’s role in promoting aerobic metabolism through the regulation of the Krebs cycle.

There are several limitations inherent to this meta-analysis. First, the total sample size ‒ 520 patients across 9 trials ‒ is relatively limited. This may result in insufficient statistical power for the analysis and increase the risk of Type II error. Second, 7 out of the 9 included studies were assessed as having a high risk of bias or raising some concerns, indicating that the overall quality of evidence is low. In particular, many trials lacked blinding or had deviations from intended interventions and outcome measurement biases; these issues may exaggerate the apparent effects of thiamine. Third, most of the included RCTs were single-center studies, which introduces potential selection bias and limits generalizability. Fourth, the included trials exhibited variability in patient inclusion criteria, thiamine administration regimens, and the proportion of patients with thiamine deficiency, leading to clinical heterogeneity. Finally, the subgroup analysis focusing on patients with thiamine deficiency was exploratory, based on extremely limited data, and lacked an adequate sample size. This precludes the ability to draw causal inferences and carries a risk of false positives.

## Conclusion

This meta-analysis preliminary implies that thiamine may have a potential effect on enhancing organ function among patients with sepsis and septic shock, as evidenced by the observed reductions in RRT utilization and 24 h lactate level, as well as larger 24 h SOFA score change. However, no significant benefit of thiamine in reducing short-term mortality was observed. It is important to note that the total number of patients included in this meta-analysis is relatively small, which, to an extent, limits the statistical power and external validity of the results. Future well-designed studies with larger sample sizes are warranted to further verify the potential therapeutic value of thiamine in this patient population.

## Funding

Supported by Jiaxing Key Discipline of Medicine – Emergency Medicine (2023-ZC-004), Jiaxing Public Welfare Research Program (2023AD31057), Jiaxing Key Discipline of Traditional Chinese Medicine Surgery of Traditional Chinese Medicine (2023-ZYYCX-002) and Jiaxing Science and Technology Plan Project (2023AD31046).

Data availability statement

The datasets generated and/or analyzed during the current study are available from the corresponding author upon reasonable request.

## Declaration of competing interest

The authors declare no conflicts of interest.

## Appendix Supplementary materials

Image, application 1

## Footnotes

Supplementary material associated with this article can be found, in the online version, at doi:10.1016/j.clinsp.2026.100901.
