# Supplementary material for: Efficacy of thiamine (vitamin B1) in sepsis and septic shock: A meta-analysis of randomized controlled trials
Source: Clinics (Sao Paulo). 2026 Mar 20;81:100901. doi: 10.1016/j.clinsp.2026.100901 (PMC13019988; doi:10.1016/j.clinsp.2026.100901)

**CLINICS-D-25-01163**

**Supplemental Material**

**Table S1** Results of Egger’s test for all outcomes.

| **Outcomes** | **p for Egger’s test** | **Publication bias** |
| --- | --- | --- |
| Short time mortality | 0.781 | No |
| Proportion of RRT | 0.477 | No |
| 24-hour lactate level | 0.744 | No |
| 24-hour lactate change | 0.538 | No |
| 24-hour SOFA score change | 0.367 | No |

**Figure S1** Sensitivity analysis for short time mortality.


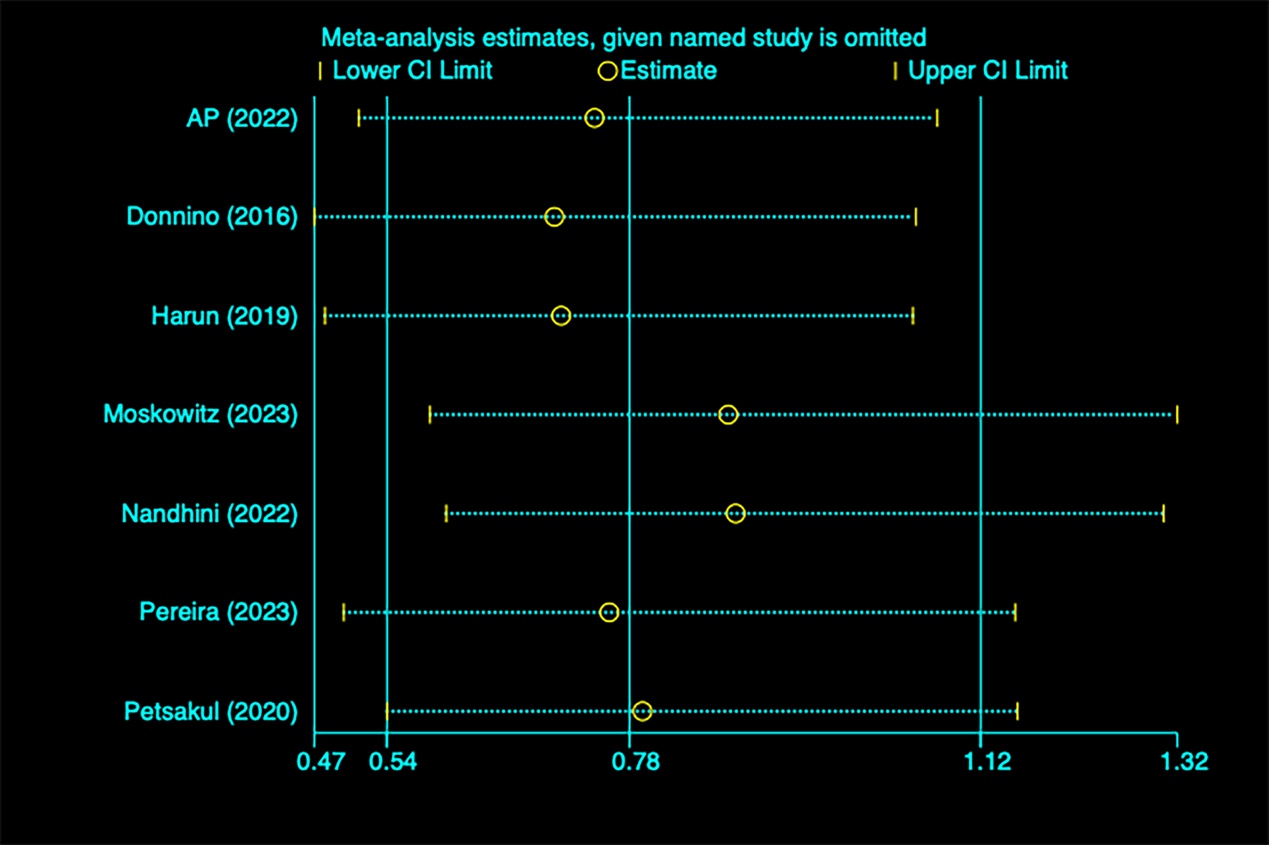


**Figure S2** Sensitivity analysis for the proportion of renal replacement therapy.


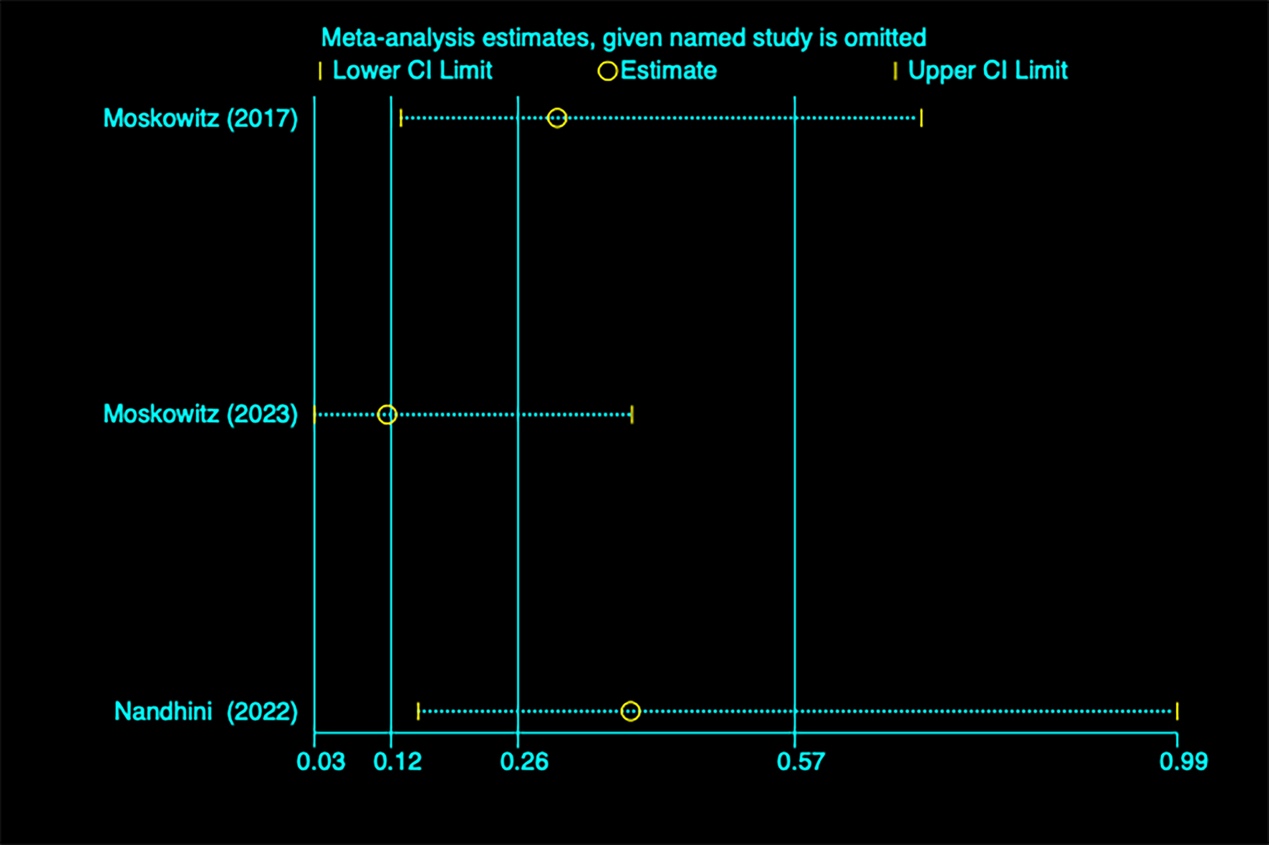


**Figure S3** Sensitivity analysis for the 24-hour lactate level.


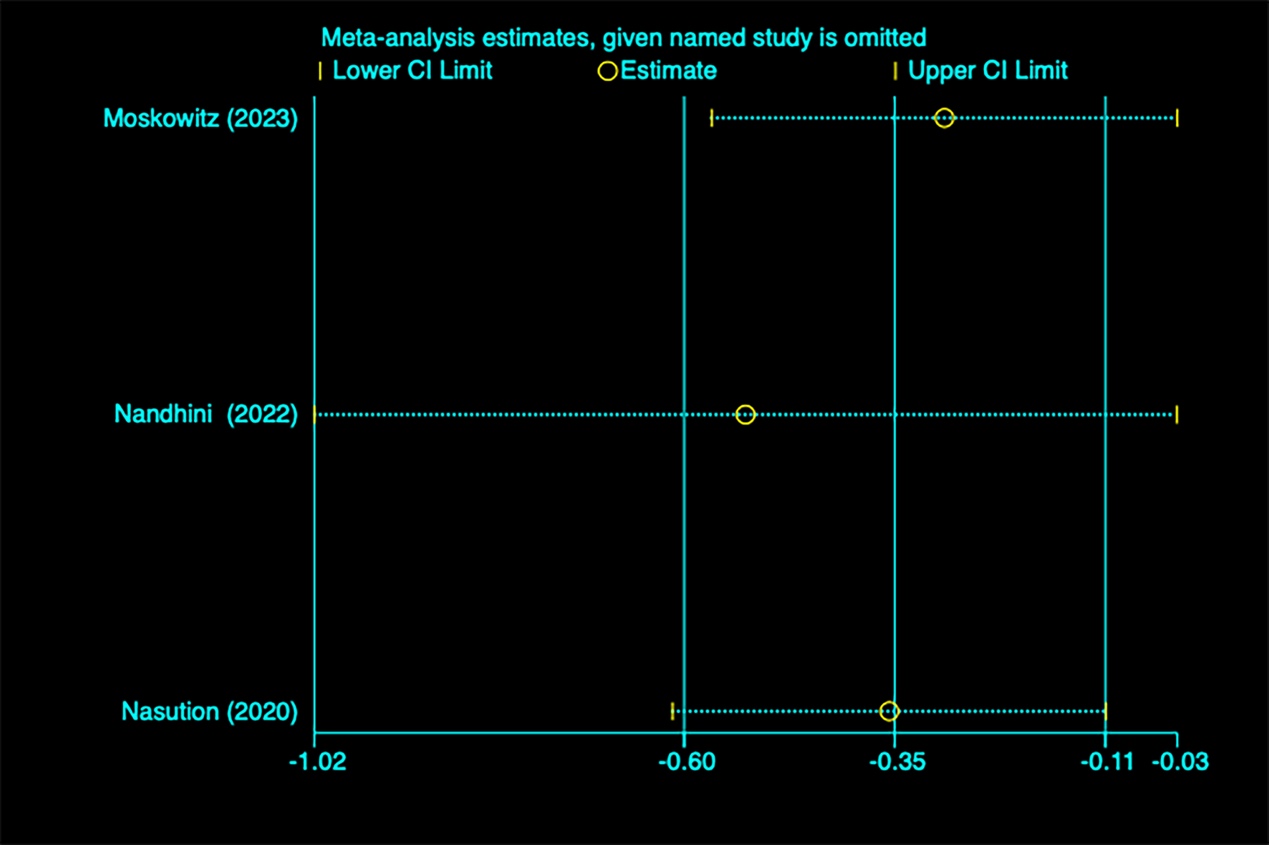


**Figure S4** Sensitivity analysis for the 24-hour lactate change.


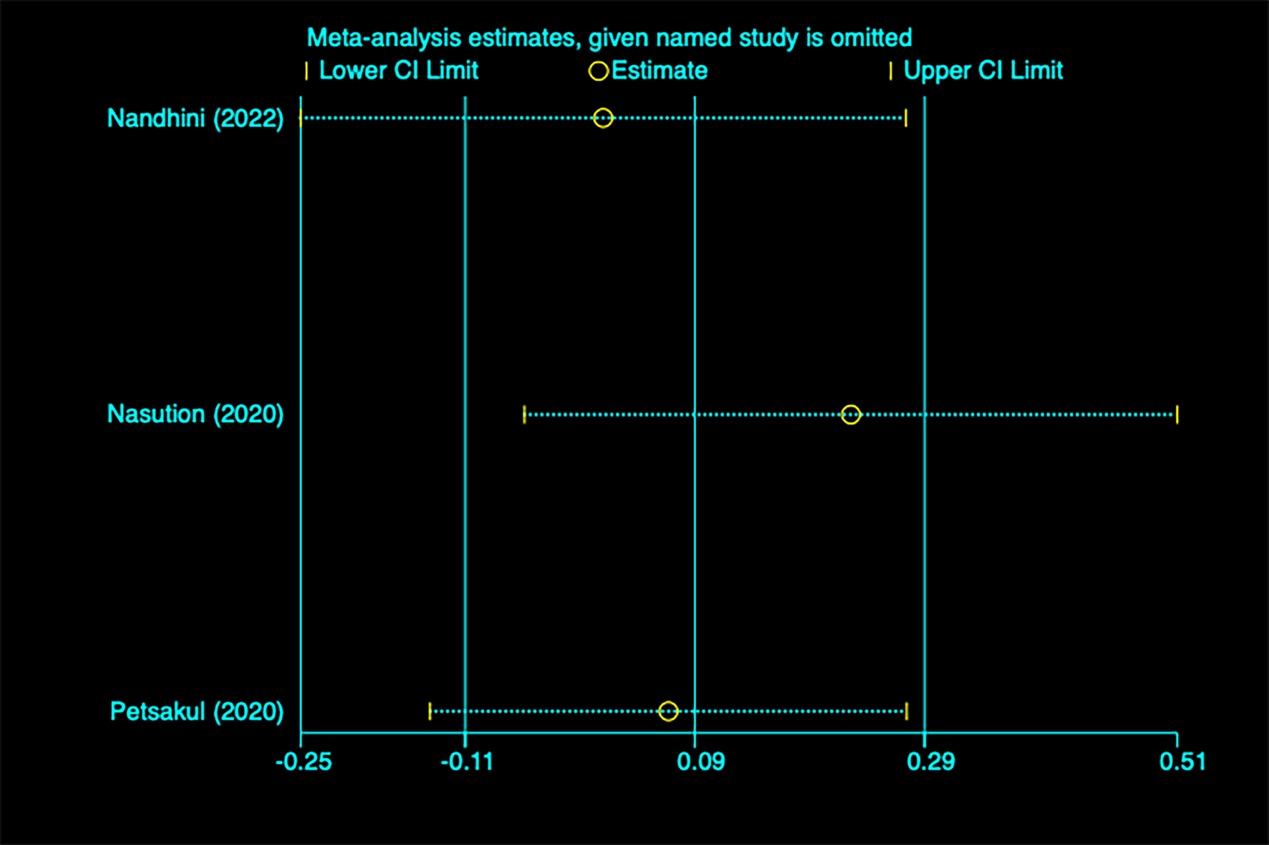


**Figure S5** Sensitivity analysis for the 24-hour sequential organ failure assessment change.


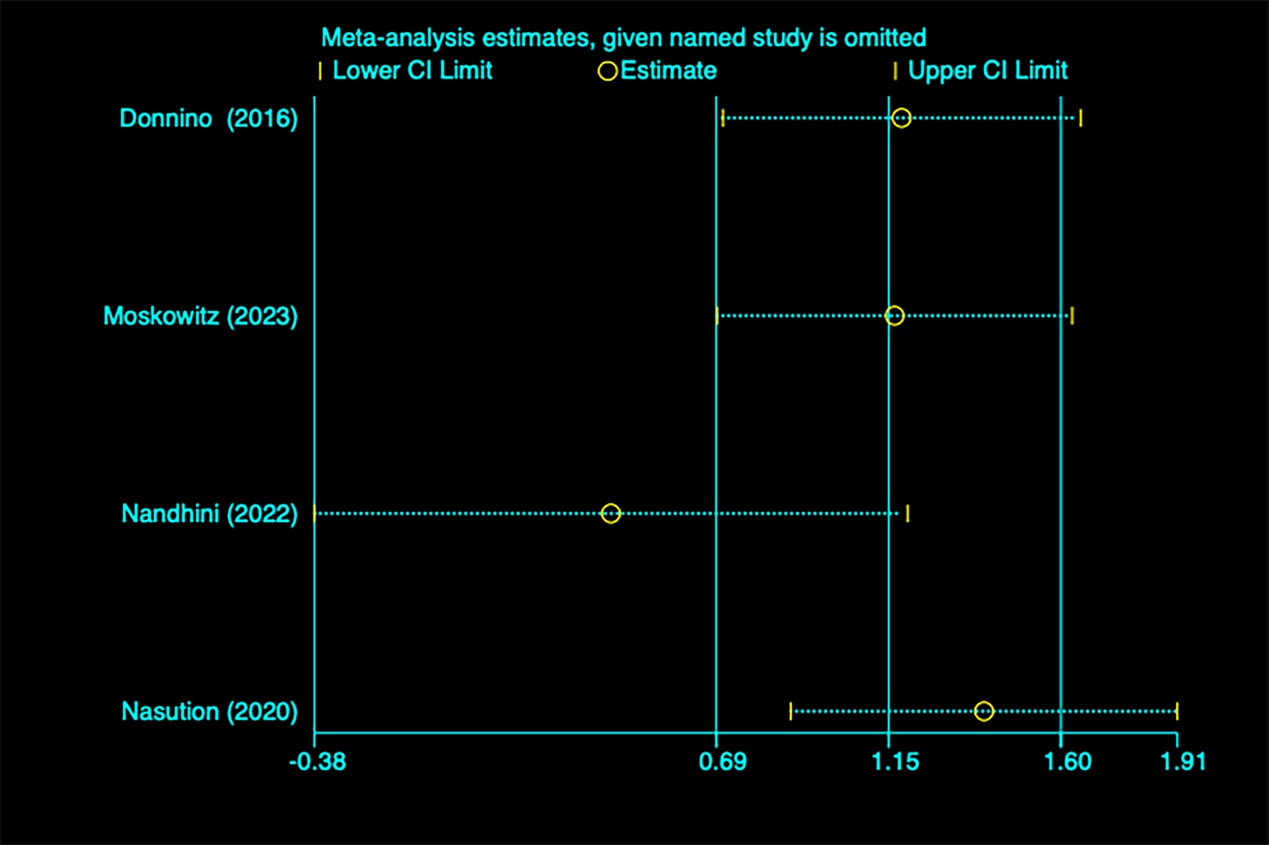

Supplement: Supplementary file 1 [file mmc1.docx]
